# Exploring the processes and mechanisms by which nonprofit organizations orchestrate global innovation networks: A case study of the COVAX program

**DOI:** 10.1016/j.heliyon.2024.e27098

**Published:** 2024-03-02

**Authors:** Hongming Xie, Manman Guo, Yingnan Yang

**Affiliations:** a Guangzhou University, School of Management, China; b Zhejiang University, College of Civil Engineering and Architecture, China

**Keywords:** Nonprofit organization, Global innovation network, Network orchestration, Public health, COVAX

## Abstract

In cocreating value with other organizations, nonprofit organizations may face multiple management challenges, posed by multistakeholder global innovation networks. Since these have not yet been systematically studied by academics, this study explores how nonprofit organizations can promote the cocreation of value in multistakeholder global innovation networks. Adopting a longitudinal single-case study approach from the perspective of network orchestration theory, this work deeply analyzes how nonprofit organizations can promote the evolution of the global innovation network of the COVID-19 vaccine under the COVAX program. The results show that nonprofits need to successively address the dilemmas of legitimacy, direction, and heterogeneity in constructing global innovation networks and that to solve these stage dilemmas, orchestrators must successively function as network architects, liaisons, and leaders to direct the implementation of network actions using trusted, leveraged, and adapted orchestration logics. This paper further proposes a model of the orchestration process and mechanisms by which nonprofit organizations facilitate multistakeholder global innovation networks. Theoretically, this study therefore extends network orchestration theory by summarizing the mechanisms and orchestration logics by which NPOs construct and develop networks when they act as orchestrators. From a practical perspective, this study also provides guidance for future unexpected global public health crises, improving the global community's ability to combat them.

## 1 Introduction

With the rapid growth of global markets, product innovation is increasingly dependent on the integration of complex technologies in multiple locations worldwide and on value cocreation achieved through multistakeholder interaction. In multistakeholder innovation networks, different actors have different resources, aspirations, perspectives and expertise [1], and they need to collaborate to create innovative solutions to complex problems to achieve ‘collaborative advantage’ [2]. Research has shown that running a multistakeholder innovation network often requires a hub actor, or a network orchestrator, who assists in creating value in the network and can coordinate multistakeholder value creation activities to facilitate or promote such value creation [3]. Network orchestrators create and extract greater network value by developing a strategic vision for the network [4], providing participants with the necessary resources for collaboration [5], and creating legitimacy for network activities [6]. When network orchestrators are third-party facilitators, they can use their leadership and resources to bring about better collaboration in organizational networks [7] and in turn derive some value from the relationships they create, i.e., value appropriation, such as innovation broker [8]. Another situation is the role of third-party facilitators who do not directly derive commercial benefits from innovation networks, i.e., value-independent network orchestrators, such as nonprofit organizations [9]. As typical value-independent organizations, the neutrality of nonprofits enhances their legitimacy in working with businesses, governments, or other types of organizations. In addition, the inherent social mission and philanthropic nature of nonprofits improve their level of credibility in the building of network relationships. Compared to for-profit organizations, nonprofits are more conducive to resource pooling and network building. However, existing research has not systematically examined how value-independent nonprofit organizations build and facilitate the cocreation of value in global innovation networks.

The COVAX program represents a typical example of a nonprofit organization building a global innovation network; it is also the representative case chosen for this study. The COVID-19 outbreak of late 2019 has caused serious political, economic and medical challenges in countries worldwide, and these countries have sought to achieve herd immunity through vaccination to escape the impact of the epidemic on the productive lives of their societies [10]. For all countries to share equal rights and obligations regarding the development, manufacture and distribution of new vaccines, three nonprofit organizations, the Global Alliance for Vaccine Immunization (Gavi), the World Health Organization (WHO) and the Consortium for Epidemic Preparedness Innovation (CEPI), have jointly led the COVAX program and established a special unit to organize its implementation [11]. COVAX provides an exchange platform for vaccine development and vaccine procurement entities, which assumes the dual function of promoting efficient vaccine development and ensuring fair vaccine distribution [12]. The nonprofit organization team is neither the manufacturer of the vaccine nor the beneficiary of the vaccine, nor does a nonprofit have statutory control over the various stakeholders, and vaccine development is cross-border, cross-regional, multisubject and highly complex [13]. Therefore, how does COVAX orchestrate the activities of various vaccine developers, funders and vaccine manufacturers in the shortest possible period and in the face of many unfavorable factors? This can be abstracted into the central question of our proposed study: “How can nonprofit organizations contribute to the cocreation of value in multistakeholder global innovation networks?”

In light of the practical demands and research gaps identified, this study focuses on the COVAX program as its research subject. To construct a theoretical model of the process and mechanism by which nonprofits promote the formation of global innovation networks, a longitudinal single-case research approach is employed from the perspective of network orchestration. This paper explores the following three sub questions: What are the roles and functions of nonprofits in global innovation networks? What is the evolutionary process of the key mechanisms by which nonprofits orchestrate global innovation networks? What is the orchestration logic through which nonprofits achieve network value cocreation? By reviewing the key events that have occurred since the implementation of COVAX, the network actions of nonprofits as orchestrators are summarized, and the important roles played by nonprofits in its processes, from network formation to network maintenance, are described.

The research contribution of this paper is therefore twofold. On the one hand, this study enriches network orchestration theory by analyzing the processes and mechanisms, nonprofit organizations, as orchestrators, build and develop multi-interest group innovation networks. Second, this study provides practical insights into global public health emergencies. That is, it summarizes a set of actionable mechanisms for nonprofit organizations to orchestrate global innovation networks and proposes a program for the design, management, and purposeful promotion of global innovation networks. Hence, this work not only provides lessons for the subsequent research and development of COVID-19 vaccines but also provides insights into vaccine research and development, enabling relevant companies to fully utilize the operational mechanism of COVAX and carry out related activities.

## 2 Literature review

### 2.1 Value creation processes and challenges in innovation networks

The innovation process among organizations does not necessarily take place within a single organization or industry but is, increasingly, widely distributed among network participants with different, complementary capabilities [1], as no single organization possesses all of the technology, knowledge, or other resources needed to innovate [14]. In this context, innovation networks have become an important innovation pathway for organizations such as firms or research institutes, which exchange knowledge and resources by constructing and developing networks throughout the innovation process, from product design to commercialization. The heterogeneity of network members brings different knowledge, logic, rights and capabilities to the focal innovation, increasing the available resources as well as the probability for the success thereof [15,16].

Innovation can be achieved through cross-organizational collaboration in a wide range of network environments; it also poses significant management challenges for the hub organizations that build the networks. That is, network hub organizations face legitimacy dilemmas in networks built around new activities [6]. Accordingly, it is imperative that external potential participating partners perceive the correctness of a particular network action. Moreover, hub organizations need to spend a great deal of time and resources early in the life of a network, communicating with different potential partners about network activities and their collective commitments. Conversely, there are different levels and natures of participants in innovation networks [4], e.g., hub firms, innovation intermediaries, or peripheral organizations that build them, which differ in terms of their innovation goals, historical cultures, and interest orientations [17]. Fighting against the heterogeneity of network participants may upset the network's equilibrium, leading to a decline in its leadership position or even a failure of innovation [18,19]. Consequently, traditional network management is gradually being replaced by "orchestration".

### 2.2 Network orchestration

The emergence of network orchestration theory has fostered new ideas for addressing collective gains at the network level. Network orchestration theory is often defined as a theory describing a set of deliberate, purposeful actions taken by a hub organization as it seeks to create and extract value from the network [3]. This theory can also be understood as elucidating the process by which a hub organization constructs and manages a cross-organizational network to achieve a collective goal [6]. The hub organization takes on a leadership role through the prestige and power conveyed by holding the central location in the network and aggregating the dispersed resources and capabilities of the network members. Research on network orchestration has focused on both the building and orchestration of networks [16], on the one hand, constructing networks can provide structural advantages to the network's orchestrators, and hub firms can change the relationships and structure of the network by choosing partners, thus controlling their network location to maintain their network centrality and status. On the other hand, the practices performed by the hub organization of a network form an orchestration mechanism, and this mechanism and its underlying practices may impact the position of the hub organization relative to other network members over time [20,21].

The actors that perform orchestration actions in a network are often referred to as orchestrators [22], and to establish and maintain appropriate structures in a network, orchestrators usually play different roles at different specific times to perform specific network activities and ensure the smooth functioning of key undertakings in the innovation network. The network actions taken by different types of orchestrators vary [23]. For example, a value-occupying orchestrator may act as a leader, directing the value-creating activities of network members, while a value-independent orchestrator may prefer to act as a liaison to facilitate the identification of innovation opportunities independently by network members [22]. While earlier studies have provided various insights into the types and roles of such orchestrators, our study attempts to closely examine the evolving role of value-independent orchestrators and the orchestration process from a process perspective under a coherent conceptual framework.

### 2.3 Value-independent nonprofit organization

Innovation typically occurs in large networks that are formed by hub organizations, which play a large role in coordinating the business relationships among network partners and mobilizing additional resources for innovation [24]. As third parties facilitating the formation of business relationships in networks, hub organizations can apply their leadership and resources to help those SMEs that lack sufficient relationship skills or beliefs to collaborate, or they can create new collaboration opportunities by providing a forum. Hubs either derive value from the relationships that they create (value-appropriation) or have no direct business interest in the relationships that they facilitate (value-independence) [7]. Innovation mediation [21] is central to studying the role of network value appropriators. Such value-appropriation type organizations are not only active participants in the collaborative creation of network value but also beneficiaries of network resources. They use their orchestrated networks to enhance their competitive advantage and profitability, achieve relatively short-term profitability through their strong resource base, advance prominent personal goals and a competitive orientation [22], and manage partners to maintain technical or commercial control during new product development [19].

Recent research has shown that partnerships between networks can be facilitated by third-party intermediaries, that these intermediaries do not directly capture the value of the network, and that such third parties can still act as hub organizations to guide network activities, but the value-independent nature of their implementation determines the noncompetitive orientation of the network activities thus engendered. The main task of these value-independent hubs is to gather the dispersed network members and support them in their independent search for innovation opportunities [24]. Therefore, as this requires exhibiting a sense of neutrality, they do not derive direct commercial benefits from the network, which makes it easier to build a common identity and develop trust in the network. This trust and common identity can accelerate the willingness of network members to share their knowledge or other resources [25]. Nonprofits are a typical example of value-independent organizations, and nonprofit organizations possess the characteristic of neutrality, therefore, some companies are more willing to establish partnerships with nonprofits to gain access to technology, knowledge, or legitimacy [7]. Nonprofit organizations rely on various sources of funding, such as donations, government funding, or business activities, to fulfil their mission and ensure the organization's continued existence. Research has shown that nonprofits can use their social mission to improve access to network resources, and the philanthropic nature and social mission of nonprofits enhance the credibility of the network relationships that are embedded in resource access. Therefore, nonprofit organizations are more conducive to building collaborative relationships, aggregating network resources, and facilitating network construction and development, but the existing research does not delve into the issue of how value-independent nonprofit organizations form and develop networks.

In summary, the literature has provided insights into the processes and mechanisms by which orchestrators form and develop networks, establishing a foundation for researchers to explore orchestrator roles and their associated mechanisms of action [21]. Successful orchestration involves network hub organizations, which influence network members by leveraging their own resources, capabilities, and relationships [20] and accumulate resources such as knowledge or technology through continuous learning [26]. However, since the literature remains more concerned with hub organizations that function as network orchestrators, typically large corporations with certain resource bases and rights [16], there is a lack of attention toward and clarity regarding the following: how value-independent nonprofit organizations orchestrate large-scale networks, the salient differences in the network orchestration process, and the driving factors behind therein. Therefore, this study explores the processes and mechanisms through which nonprofit organizations orchestrate global innovation networks with a specific focus on nonprofit organizations.

## 3 Research design

### 3.1 Research methodology

Case studies can provide a comprehensive understanding of complex social phenomena and are suitable for answering the "how" and "why" questions regarding such phenomena. Because little is known about the processes through which nonprofits build, maintain, and facilitate networks, this study has chosen to adopt a longitudinal single-case study approach, focusing on the dominant actions of nonprofits in networks and the evolution of such networks. The longitudinal single-case study approach is based on the following considerations. On the one hand, the purpose of the study is to construct a theory to explain how a particular phenomenon emerges, evolves, and terminates over time. From a process perspective, we assess the same case from multiple time points, which is conducive to confirming the sequence of key events and clarifying the interaction mechanisms between different actors. Assessing such dynamic processes over time using a longitudinal single-case study approach is worthwhile [27]. On the other hand, applying a longitudinal single-case study approach can deconstruct the complex actions of the research subjects in multiple contexts, which is conducive to exploring the logical and causal relationships among the constructs in the critical temporal evolution process and facilitates the subsequent process model construction.

### 3.2 Case selection

We chose COVAX, the COVID-19 Vaccines Global Access program, because the case demonstrates the evolutionary process of a unique nonprofit global innovation network. Since the outbreak of COVID-19, the lives of people worldwide have been seriously threatened. The only way to stop the spread of the virus is to develop a vaccine and achieve herd immunity; however, due to the lack of knowledge about COVID-19 and the vaccine development technology being scattered worldwide, many countries without any experience or therapeutic drugs have started to develop a COVID-19 vaccine. Past experience with the treatment of influenza H1N1 shows that vaccine development requires significant capital investments and comes with a risk of failure [28,29]. Moreover, the R&D, production and procurement of vaccines are usually monopolized by companies or other organizations in most developed countries. The countries to which these companies or organizations belong use advance purchase agreements to stockpile doses before production, severely limiting the doses and timing of the vaccinations available to developing countries. To avoid the prevalence of vaccine nationalism and to address the inequitable distribution that prevents countries with low-income economies from being vaccinated, there is an urgent need for an impartial organization linking stakeholders, such as governments, vaccine development manufacturers, foundations and investors worldwide, to address the twin issues of promoting vaccine development and equitable distribution. As a result, the WHO led Gavi and CEPI to jointly establish the COVAX program.

Under the COVAX pillar, each organization has a main responsibility related to equitable access. CEPI, for instance, has the primary responsibility for creating an R&D portfolio of COVID-19 vaccines and ensuring that the vaccine developer commits to allowing COVAX to be a ‘first-choice’ in procuring vaccines developed through partnering agreements [30]. The procurement arm of COVAX is overseen by Gavi and manages global procurement and the deployment function for COVID-19 vaccines for participating countries of all income levels. Gavi is also responsible for the COVAX Advanced Market Commitment, which supports the participation of low- and middle-income countries in COVAX. The WHO, among its many responsibilities, establishes the principles of fair allocation, which ultimately form the basis for the recommendations and decisions related to the allocation of vaccines to countries participating in COVAX.

The main objectives of COVAX are to provide financial support for promising vaccine candidates, stimulate venture capital investments in manufacturing capacity and ensure equitable worldwide access to vaccines. However, multiple challenges and dilemmas were faced during implementation [10], the first of which concerns program acceptance and international politics [31]. Specifically, there have been doubts or concerns about the fairness and enforceability of the COVAX program in certain countries and regions, resulting in low vaccination rates. This poses an obstacle to the global immunization strategy, as the purchase and distribution of vaccines is influenced by political factors and international relations in each country, thus hindering the achievement of COVAX goals [32]. The second issue concerns funding shortfalls; despite multinational commitments to funding, the COVAX program continues to be underfunded, but meeting the global demand for vaccines still requires significant funding to purchase and distribute them. The third issue is vaccine shortages [10]; when a COVID-19 vaccine was first introduced, many developed countries had already signed purchase agreements with pharmaceutical companies, resulting in both national and global vaccine supply shortages [33]. Hence, the COVAX program has struggled to obtain sufficient quantities of vaccine, even when funding is available.

However, from a practical point of view, the three nonprofit organizations—Gavi, WHO and CEPI—are not R&D actors or recipients, do not have a strong economic base and cannot control resources, and face numerous challenges in achieving the objectives of COVAX, such as how to coordinate stakeholders, how to promote vaccine development or how to establish a distribution system. This study has chosen to examine COVAX to help understand the central question of “how nonprofit organizations can facilitate multistakeholder global innovation networks to create value together.”

### 3.3 Data collection

Data collection method in this study involved the text collection method, mainly to obtain exhaustive secondary data. The data collected for this study start from January 1, 2020–September 30, 2023. COVAX is a global program involving many regions and industries. In recent years, the issue of vaccination and vaccine distribution has received extensive attention in many countries, and the number of organizations involved in vaccine development has continued to increase. Thus, there is a wealth of relevant secondary data, as shown in Table 1.

**Table 1:** Text data sources.

Hub Actors	Data Content	Amount	Data source
Gavi	Press Releases	69	https://www.gavi.org
	Documents	21	
	Quotes of Leader Interview	66	
WHO	Press Releases	16	https://www.who.int
	Documents	25	
	Quotes of Leader Interview	8	
CEPI	Press Releases	64	https://cepi.net/covax
	Documents	3	
	Quotes of Leader Interview	28	
Papers		43	Web of Science

First, we obtained 149 official press releases from the official websites of Gavi, the WHO, and CEPI, which encompassed the full range of specific events that have taken place since the establishment of the COVAX program regarding the development of coordinated and distributed vaccines by these three organizations. We also extracted a total of 102 leader interview quotes from official press releases to create an initial database of the COVAX program.

Second, we obtained a total of 49 position papers and public statements related to the guiding principles, medium-term projections, and strategic planning of COVAX from the official websites of Gavi, the WHO, and CEPI to complete and supplement the initial database. The compiled textual content comprises roughly 310,000 words in English.

Finally, multiple data sources allowed comprehensively examining the focal issue while enhancing the database's persuasive and explanatory power. Hence, this study cross-checked the collected secondary data with the published papers and supplements to improve the credibility of the case study. Specifically, we searched for "TS=COVAX" on the core set of Web of Science and retrieved 224 papers. After reading and screening each paper, we obtained a total of 43 high-quality papers.

### 3.4 Data analysis

Data analysis entailed iteratively comparing the activities of orchestrations' practices with theories of network orchestration to analyze the actions or events at a given time by identifying key events and constructing clear time stages. Specifically, this analysis was divided into four steps:

First, key event identification was performed. The key events referred to events that were significant and had far-reaching impacts on the subsequent implementation of the focal program. By assessing the official press releases, official documents, and quotes with leaders interview of Gavi, the WHO, and CEPI, this study cleansed the information, organized the events with the collected secondary data, and cross-checked the secondary data for mutual corroboration and complementation, thereby improving the credibility of the case study. Ultimately, a reliable event database was formed.

Second, the event stages were categorized. The examined data were sorted chronologically by the key COVAX events that have occurred since its inception and were based on the key nodes derived from dividing the COVAX program into three phases to track the changes that occur between phases. The network construction phase began in June 2020 with the first launch of the Vaccine Advance Market Commitment (Gavi COVAX AMC) at the Global Vaccine Summit hosted by Gavi and the UK, marking the formal establishment of COVAX; the network operation phase began in November 2020 with the first COVAX committee meeting, marking the program's transition from design to implementation; the network maturity phase began in February 2021, when the first vaccine doses were shipped to Ghana, marking the start of the global rollout.

Third, the initial conceptualization was established. This study adopted inductive thematic analysis, following Gioia et al. (2012) to code the initial data and abstract the concepts to rigorously implement this analysis inductively and, finally, form the theoretical themes [34]. As this study unfolded in three main phases, specific coding was implemented according to the time series strategy. The key events were independently analyzed and compared to the literature on network coding, with a view toward the emerging initial first-order constructs. The core events of COVAX's network formation or orchestration in different phases were then compared to find the similarities and differences among the first-order constructs, which were compared to the literature to form multiple, rigorous second-order themes. To improve reliability and validity, the research team members first conducted independent analysis, discussed any inconsistent results and then finally formed a consensus (Appendix). Taking the stage of global innovation network construction as an example, according to Perks et al. (2017), hub organizations need to enable potential partners to understand the potential value of network cooperation and deepen their cooperation with enterprises or projects to enable them to participate in the focal network action together during the arrangement of the focal network. This study thus first derived first-order concepts such as "organizing a global summit" and "issuing a global call for proposals" via matching. Then, from a theoretical perspective, second-order themes with theoretical connotations were abstracted based on these identified first-order concepts. For example, "organizing a global summit" actually aims to lead potential partners to envision the advantages of participating in network cooperation, consequently, it was refined and categorized as a second-order theme, "envisioning".

Finally, a theoretical framework was proposed. Based on the emergent constructs and themes, this study further aggregated the core concepts into dimensions, and the formation of these aggregated dimensions mainly followed existing research on network orchestration. For example, the results of this study indicated that orchestrators [22] may adopt different sets of practical actions when forming, sustaining, or developing a network and that each set of such actions forms mechanisms that may change the orchestrator's position relative to other network members over time [14]. However, it remains unknown how an orchestrator can implement the appropriate orchestration mechanisms depending on the stage of the dilemma they are in. In this study, after repeatedly confirming the characteristics of correlation, explanatory power, and reproduction frequency among the different dimensions and across the data sources, research topics, and literature, a robust logic of correlation was formed. Thus, we could finally propose the theoretical framework of a process evolution model of orchestration of global innovation networks by nonprofit organization based on network orchestration theory.

## 4 Research findings: The operational mechanisms behind innovation networks and their evolution

The main purpose of this study is to provide new insights into how nonprofit organizations can contribute to the construction and development of global innovation networks. In this section, we describe the findings in the network construction phase, network operation phase, and network maturity phase in detail. We explain the main dimensions and constitutive themes of each stage, including network context and orchestrator roles, orchestration mechanisms and orchestration outcomes, and we analyze the interactions among them.

### 4.1 COVID-19 vaccine global innovation network construction phase (June–November 2020)

Network background and orchestrator role. In early 2020, COVID-19 virus enters a state of rampant transmission. Driven by national security or economic interests, organizations such as governments, enterprises and universities carry out COVID-19 vaccine research and development according to their own technical capabilities. For example, Modena (USA) and BioNTech (Germany) chose the nucleic acid vaccine preparation method, while China National Pharmaceutical chose the traditional inactivated vaccine preparation method. Knowledge, R&D technology and capital support for COVID-19 vaccines are characterized by a relatively decentralized situation, where each company and research institution have its own technology and resources for vaccine development and these assets are locked within the boundaries of each organization. This fragmentation may have implications for the global process of herd immunization, as it fails to take full advantage of the synergies that can be achieved by working together and may lead to relatively slow vaccine development and dissemination.

To pool global vaccine R&D resources and accelerate the end of the pandemic, Gavi, WHO and CEPI jointly led the COVAX program and invited relevant organizations to participate, and a multistakeholder global innovation network for the COVID-19 vaccine was formally initiated. In this study, we refer to the leadership teams of these three nonprofits as the "orchestrators" of the global innovation network for COVID-19. At the inception of the program, potential participating members did not have sufficient trust in the COVAX program, many large vaccine developers were concerned about sharing clinical or trial data, and the participating countries questioned the fairness of vaccine distribution; the principal dilemma facing the orchestrators was legitimacy. Legitimacy refers to the conformity of an orchestrator's network activities with the social values held by a broad audience; it can only be established when potential participating members perceive the orchestrator's actions as the "right thing to do" [6]. To resolve the legitimacy dilemma, orchestrators act as architect, building collaborative relationships among organizations [23]. The architect's role revolves around creating visions, agendas, goals, and legitimacy activities, initiating gatherings of network members, and envisioning how individual interests can be linked to shared aspirations [18]. For example, the orchestrators in the COVAX program, on the one hand, invited organizations to join the network, together, by entering into preferential or funding agreements. On the other hand, multiple incentives were developed to stimulate governments or businesses to join the COVAX program in terms of building the interest and support of potential network members for future activities.

Orchestration mechanism. To attract more organizations to COVAX, orchestrators connect with potential participating members in two primary ways. The first is the envisioning mechanism (Perks et al., 2017), in which orchestrators work to envision the value of the network for potential members and help them understand that vaccine development can be accelerated through the development of collaborative relationships among all parties [20]. For example, the orchestrators cohosted a Global Vaccine Summit with the United Kingdom, which involved 62 countries, the United Nations and its related organizations, the pharmaceutical industry, the private sector, foundations, and other partners. Gavi CEO Dr. Seth Berkley explained to the public Gavi's important role in shaping the global vaccine market to ensure equitable access to vaccines, stating "One thing that has been made all too clear over the past few months is that this disease does not respect borders, which is why this global problem requires a global solution". The main objective is to explain the role of the COVAX program, which is to lead the country, companies or related organizations into understanding the purpose of the COVAX program and to mobilize the participation of public organizations.

The second key action is to connect, which proceeds by bringing together related organizations to develop specific networking actions [6]. Due to the urgent need to attract many organizations in the early stages of COVAX's establishment, its orchestrators attracted different types of organizations to participate by, for example, issuing a global call for proposals. In this example, to increase support for the COVAX core working group, the orchestrators issued a global call for proposals and selected 10 civil society organizations to join the core working group based on a range of criteria, including technical expertise, civil society organization experience, and immunization knowledge and technology. The CEPI orchestrator, on the other hand, who is committed to building a vaccine R&D portfolio, selected R&D partners in a targeted manner, evaluated vaccine candidates based on the criteria of its global R&D labs, and selected candidates with the potential to facilitate a successful launch.

Orchestration Outcome. During this phase, potential participating members gradually built-up trust in the orchestrator, and several countries or economies, including the EU and China, opted to join COVAX, including COVAX AMC participants. In addition, the orchestrator selected 10 civil society organizations from around the world to participate in COVAX core working groups, such as the inclusion of the International Rescue Committee in the COVAX Coordination Meeting Group, thus providing the necessary support to promote political and community engagement. At the same time, the CEPI orchestrator entered into collaborative R&D or manufacturing agreements with several vaccine R&D laboratories or vaccine manufacturers, such as SK bioscience in Korea and Biofabri in Spain, to provide financial support for vaccine laboratories with R&D capabilities to accelerate the pursuit of COVAX vaccine portfolio goals. The orchestrators were able to engage potential network members and initially develop the ability to establish and coordinate collaborative relationships between organizations in this phase.

### 4.2 COVID-19 vaccine global innovation network operation phase (November 2020–February 2021)

Network background and orchestrator role. At the end of 2020, several leading companies worldwide rated the development of a COVID-19 vaccine and entered phase III clinical trials, thus facilitating the operational stage of the COVAX COVID-19 vaccine global innovation network. The orchestrator, the WHO, included vaccines on the Emergency Utilization List (EUL) that had been evaluated for eligibility, but only two vaccines, Oxford/AstraZeneca and Pfizer BioNTech, were on the EUL during this stage, and capacity was still far short of the needed number of global doses. Orchestrators continued to seek partnerships with vaccine laboratories with R&D potential to rapidly develop new coronavirus vaccine portfolios. However, limited financial resources required orchestrators to select targeted partners. How to accurately and efficiently identify the laboratories with the highest potential for vaccine development and ensure that the vaccines they have invested in would have value in the future was a difficult problem. Accordingly, the network of orchestrators faced the dilemma of targeting.

To balance each value-creating activity and truly invest limited resources in vaccine R&D efforts with growth potential, orchestrators acted as liaisons during this phase [35], beginning to purposely seek out potentially valuable members, evaluate vaccine effectiveness by creating collaborative workgroups, and continually adjust internal R&D structures to fit this phase. For example, during collaboration with a vaccine candidate that had been developed at the University of Queensland, problems with certain interfering diagnostic tests meant that the vaccine was not suitable for widespread use, therefore, the orchestrator chose to stop funding further development of that vaccine. The orchestrator acts as a liaison during this phase, coordinating interorganizational interactions and handling the entry and exit of members from the network.

Orchestration mechanism. During this phase, more vaccine candidates entered phase III clinical trials; to ensure that the companies likely to be successful in developing vaccines in the future were prioritized to sell vaccines to COVAX and use them for global distribution, the orchestrators gradually built up the mechanism of innovation appropriation. Innovation appropriation is a mechanism that ensures that all participants have a fair share of the value of their network [14]. Hence, the orchestrators booked and appropriated a portion of the innovation capacity of vaccine developers or vaccine manufacturers in advance by expanding their existing partnerships. For example, the CEPI orchestrator added $5 millions of available funding to expand the vaccine manufacturing process at vaccine manufacturer Biological E. CEPI's CEO, Richard Hatchett, noted that "Bio E's vaccine candidate has the potential to be produced at scale, and characteristics which could make it suitable for broad distribution in developing countries".

At this point in the epidemic, any laboratory development could have provided a new opportunity to fight the virus. To ensure that everyone benefits from orchestrator funding [36], the orchestrators chose to regularly release data from collaborating laboratories during the maturation phase of the COVID-19 vaccine. This knowledge diffusion mechanism for sharing, acquiring, and deploying decentralized knowledge serves to accelerate the development of new or variant virus vaccines [14]. For example, the CEPI and Oxford/AstraZeneca orchestrators jointly announced positive data from the Phase III clinical trial in November 2020, and these project results and R&D data provided a good start for the Oxford team, while the CEPI orchestrator provided a timely catalytic investment to fund the late-stage trials, preclinical work and manufacturing materials for this vaccine candidate.

Orchestration Outcome. To address the targeting challenge, coordinators continue to choose to expand collaboration with vaccine developers and producers, such as Clover Biopharmaceuticals, Ltd. or the Indian vaccine manufacturer Biological E. Ltd., among others. Orchestrators offered high levels of funding to these vaccine R&D manufacturers to attract their participation, identified future resource utilization opportunities through funding agreements or capacity agreements, ensured future innovation outputs that can be occupied by the COVAX program, and ultimately achieved the goal of developing leveraged innovation utilization.

### 4.3 COVID-19 vaccine global innovation network maturity phase (February 2021 - Present)

Network Background and Orchestrator Role. With the first vaccine doses shipped to Ghana, COVAX has officially moved into the vaccine delivery phase. The Serum Institute of India, which produces Oxford/AstraZeneca vaccines, was an early pillar in the COVAX supply chain. However, following the massive outbreak in India, its government restricted the export of vaccines, resulting in the disruption of the largest vaccine supply chain. Other suppliers who were able to deliver vaccines on a regular basis were unable to provide the full number of doses needed due to lack of capacity, consequently, COVAX faced a heterogeneity dilemma of vaccine supply shortage, with access to vaccines increasingly dependent on donations from wealthy countries. The heterogeneity dilemma refers to the inability of an organization to obtain the resources desired in a particular act of cooperation or coordination, whereas the resources that are readily available do not satisfy the demand, thereby generating the complex decision-making dilemmas faced in attempting to solve the above problem. To secure funding for further smooth operation of distribution and delivery and to address the heterogeneity dilemma, the orchestrators undertook many mobilization campaigns, calling for donations of vaccines or funds from governments or private institutions. At this point, the orchestrators took on the role of leader [18], allocating resources for activities and identifying new stakeholders, using their continuing authority to lead or maintain the network's activities while continuing to enhance their reputation and credibility. For example, after the delivery phase began, the orchestrators launched a series of major fundraising events, such as the One Protected World Summit, the Go Give One campaign and the Vax Live concerts, with the main aim of mobilizing members to participate and to secure the funds needed to secure vaccine development, manufacturing and delivery.

Orchestration Mechanism. During this phase, the orchestrators continued to host formal and informal network mobilization events. Network mobilization involves the orchestrator's encouragement of actors to participate in network activities and innovate toward a common network goal [14]. For example, the "One Protected World" event that the Gavi orchestrator cohosted with the United States brought together world leaders, the private sector, civil society organizations, and key technology partners to galvanize resources and firm up commitment to the COVAX AMC financing mechanism. Similarly, in a mobilization campaign that was primarily aimed at fundraising, the WHO orchestrator launched the Go Give One campaign on Facebook, which allowed companies from around the world to contribute through a simple fundraising mechanism offered on Facebook and individuals to directly fund access to vaccines in low-income countries through this platform. At this time, the nonprofit team was engaged in numerous interactive activities with participating members to foster relationships through online mobilization.

As part of the consultation process for developing the 2022 COVAX strategy, the Gavi orchestrator discussed the impact of the pandemic on routine immunizations and the next adjustments to COVAX's strategy to support countries in controlling the virus during a board meeting that occurred in September 2021. Alignment involves making strategic actions or collaborations in a network more explicit and adjusting current decisions in a timely manner to fit the goals of the network [20]. As Gavi's CEO Seth Berkley said, “As the global solution designed around equitable access to COVID-19 vaccines, COVAX's strategy will continue to adapt as the pandemic evolves”. For example, removing all export restrictions, holding manufacturers to their COVAX commitments and providing transparency regarding delivery schedules and vaccine delivery sequences have become their principal strategies. This enables countries that have achieved high coverage to take their place in the COVAX queue and support the low- and middle-income participants. In December of the same year, the Gavi orchestrating directors resolved to grant Gavi the authority to allocate funds for current and future COVID-19 vaccine deliveries under the guidance of the new COVAX coordination structure, an action based on emergency and urgent country needs that does not require independent review, reflecting the orchestrators' adjusting of network actions, and the establishment of adjusting mechanisms to facilitate further adaptation orchestrator actions according to the evolution of the network.

Orchestration Outcome. During this phase, the number of member countries and vaccine development manufacturers joining COVAX was largely stable, but the orchestrators still developed their initial ability to maintain network stability by adjusting mobilization mechanisms according to network needs and continuously strengthening the reciprocal relationships among key network members to maintain effective network relationships. For example, the CEPI orchestrator continues to invest an additional $36.9 million in Clover Biologics to support the development of vaccine candidate SCB-2019, which will provide up to 414 million doses of vaccine to COVAX facilities for equitable distribution pending placement on the EUL.

## 5 Case discussion: Nonprofit-led global innovation network orchestration

**Fig. 1:**
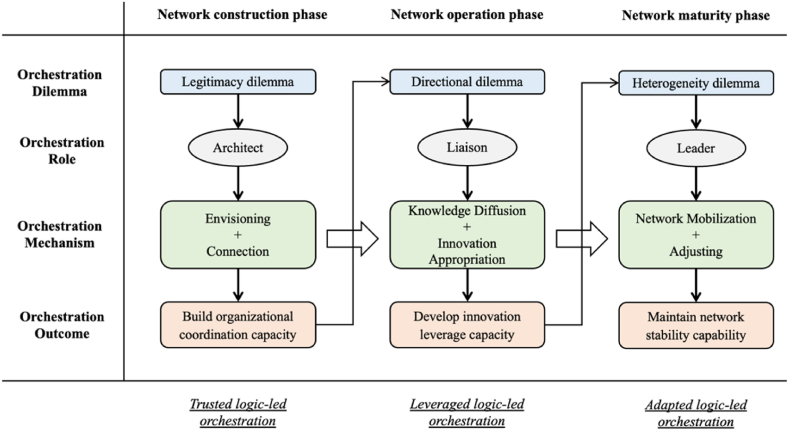
Evolutionary model of the orchestration process of the COVAX global innovation network.

Through the analysis in the previous chapter, we found that the process of constructing and developing global innovation networks by nonprofit organizations in the COVAX program is accompanied by a series of dynamically changing orchestration actions. In the face of legitimacy, directional and heterogeneity orchestration dilemmas, nonprofit organizations choose to play the roles of architect, liaison and leader to guide network members through a series of orchestration network actions. Stage dilemmas are the main reason for changes in network actions, and orchestrators need to play different roles in dilemmas to conduct network orchestration actions to enhance their capabilities in the current stage. Through the analysis of the COVAX program in the previous section, and in conjunction with the current literature on network orchestration, we have refined the portfolio of orchestration actions in the COVAX program, including envisioning, connection, knowledge diffusion, innovation appropriation, network mobilization and adjusting. When orchestrators have achieved their stage orchestration goals, further demand for innovation outputs generates the next dilemma. Based on this, we have proposed an evolutionary model of the orchestration process of the COVAX global innovation network, shown in Fig. 1.

### 5.1 Evolution of the role of nonprofits as orchestrators

In the COVID-19 Vaccine Global Innovation Network, three nonprofit organizations, Gavi, the WHO, and CEPI, formed a team of orchestrators because of their common goals. Early research on network orchestration has focused on the orchestration of central firms in large networks, emphasizing that central firms enforce power with resource control or by playing specific roles and that they meet salient personal goals and obtain relatively short-term profitability by leveraging their network to increase competitive advantage and profitability [22]. However, when the orchestrator is a nonprofit organization, its nature of not deriving value from the network dictates an inability to leverage resources and capabilities to drive network development. Not-for-profit organizations are usually not resource-rich or influential players, securing power through strong relational positions rather than monetary resources or intellectual property while conducting unofficial orchestration based on the important characteristics of neutrality and integrity. Nonprofit orchestrators are thus more concerned with collective innovation goals and noncompetitive orientations, as well as common interests such as network substance and survivability, than their own financial interests [22]. In the focal case, the emergence of stage dilemmas thus forced orchestrators to change the roles they played and appropriate roles that allowed them to better implement the appropriate mechanisms of action to solve the dilemmas faced in their current stage and to promote the development of the innovation network to achieve its goals.

Specifically, during the network construction phase, potential network members may become skeptical about the value of the nascent network. To address this legitimacy dilemma, orchestrators need to build broad interest and support from potential members for future network activities. The activities associated with the architect role also begin in the early stages of network creation. During this phase, the specific activities played by the orchestrator in the architect role revolve around selecting network participants, identifying opportunities, and setting goals. By initiating activities such as network gatherings, orchestrators can mobilize network members to identify opportunities for value creation and establish a common vision to cocreate network legitimacy [23].

During the operation phase of the network, orchestrators find that the limited available resources are no longer able to support all network members in value-creating activities, and they therefore face a directionality problem. To ensure investment in the most promising network members, the orchestrator must begin to play the role of a liaison. During this phase, the liaison's activities revolve around resource allocation, which entails identifying new stakeholders, handling member entry and exit, and enhancing relationship building commitments. Through efficient delegation and support, the liaison facilitates core network members in effectively conducting challenging activities [18].

In the network maturity phase, as the network matures, orchestrators begin to face the problem of distributing the value output of the network, and during this phase, the heterogeneity dilemma is the main dilemma faced. To resolve this dilemma, the orchestrator shifts from a liaison role to a leader role, using its ongoing authority to promote network stability. The orchestrator encourages network members to build relationships and assign value outputs or new tasks to enhance the network's reputation and credibility, thus maintaining the network's continuous and stable operation (Fig. 1).

### 5.2 Key mechanisms and orchestration logic of nonprofits as orchestrators of the global innovation network

Network orchestration follows an ongoing and evolving set of rules for action and a collection of actions that orchestrators use to structure their networks. As the structure of the network continues to change, orchestration mechanisms and their underlying practices may alter the positions of orchestrators relative to other network members. In addition, the practical actions of orchestrators follow a particular logic, value creation, which, in different stages determines the appropriate orchestration mechanisms that orchestrators engage to resolve their stage dilemmas, as well as the orchestration capabilities that orchestration acquires in constructing and developing the network in these different stages. In the focal case, the orchestrators’ orchestration logic demonstrated the stage change "trusted - leveraged - adapted", as follows:

In the network construction stage, the action mechanism of the nonprofit orchestrators follows a trust-based orchestration logic. Research suggests that the emergence of envisioning should be catalyzed at an early stage of network formation [37], as the envisioning mechanism is a prerequisite for the smooth functioning of other orchestration mechanisms [20]. Another important mechanism established by orchestrators in this phase is connection, which not only accelerates the rapid formation of global innovation networks by bringing together relevant organizations to develop specific network relationships but also enhances the confidence of existing members regarding their participation in network activities [6]. The implementation of the envisioning and connecting mechanism facilitates the formation of organizational coordination capacity, which can facilitate the connection of new members and coordination of interorganizational relationships, give full play to organizational strengths, and, ultimately, maximize the value of the network, thus assisting network members in gradually building legitimacy. Therefore, this study defines this type of orchestration combination, which guides potential members to envision the future value of the network and establish a common vision, to solve the legitimacy dilemma through a series of practical activities and to improve their own organizational coordination capacity, as trusted orchestration logic.

In the network operation stage, the action mechanisms and orchestration outcomes of the nonprofit orchestrators follow a leveraged orchestration logic. For orchestrators playing the architect role in this phase, guiding network members to share knowledge in a maturely operating network becomes the most important mechanism of action. Knowledge diffusion refers to the explicit sharing of expertise among network actors and the knowledge spillover that occurs through interaction [14]. However, knowledge diffusion presupposes the establishment of innovation appropriation mechanisms in the network for the equitable appropriation of innovations [14], and if orchestrators do not establish equitable appropriation mechanisms in the network, network members reduce the diffusion of knowledge or lock the output value within their own boundaries. The mechanisms of knowledge of diffusion and innovation appropriation together drive the emergence of the orchestrator's innovation leverage capability, which is the ability of an orchestrator to reuse or deploy to a network member's technology, process, or other innovation assets [19]. Therefore, this study refers to this combination of orchestration mechanisms, whose main purpose is to leverage the potential value of the network, to solve the directional dilemma of insufficient resources and to enhance its own innovation leverage capacity through a series of practices, as leveraged orchestration logic.

In the network maturity stage, the action mechanisms and orchestration results of nonprofit orchestrators follow an adaptive orchestration logic. In this study, as the number of people infected with the COVID-19 virus increased dramatically worldwide, COVAX needed to accelerate the process of vaccine candidate development. Accordingly, the COVAX board of directors conducted an extensive strategic alignment effort to align their existing strategic actions with the network development process. In addition, to secure additional vaccine and financial donations to address the heterogeneity dilemma, orchestrators play the roles of leaders engaged in network mobilization activities to facilitate the connection of new relationships and to match different organizational relationships [14]. Network mobilization and adjusting mechanisms can work together to promote the ability of orchestrators to maintain network stability, and the ability to maintain network stability can promote network members to engage in network innovation activities in an atmosphere of trust and openness; hence, the more stable the network is, the higher the value creation capacity [3]. Therefore, this study refers to this combination of orchestration mechanisms, which mainly aims to adapt current strategic actions to solve the heterogeneity dilemma and enhance its ability to maintain network stability through a series of practices, as adapted orchestration logic.

## 6 Contributions and perspectives

### 6.1 Research contributions

This study extends the scope of research on network orchestration theory by providing an in-depth analysis of the processes and mechanisms of nonprofit organizations, as orchestrators, in the construction and development of multi-interest group innovation networks. The literature on network orchestration initially focused on firms that are central in large networks, whose orchestration strategies are usually based on achieving their business goals [37]. These strategies mostly focus on resource control and the execution of specific functions to enhance competitive advantage and increase profitability potential, while the central firm's goals are often short-term and individually profit-oriented [22]. However, in a scenario where nonprofit organizations are orchestrators, these organizations do not primarily seek to derive direct economic value from their networks. In contrast to entities with abundant resources and vast influence, nonprofit organizations rely more on their positions in their social relationships to ensure their impact than on financial resources or intellectual property. In this context, the intermediation activities of nonprofit organizations emphasize their core values of neutrality and integrity. Such orchestrators place more emphasis on collective innovation goals, noncompetitive orientations, and related core issues and common interests within their network rather than purely pursuing financial interests. Based on this study, we suggest that when nonprofit organizations take on the role of orchestrator, their mechanisms that drive value creation among network members not only catalyze innovation effects within their network but also strengthen their own orchestration capabilities. This enhanced orchestration capabilities further fuel the innovation activities of nonprofit organizations in subsequent stages. In addition, this study has summarized the "trusted-leveraged-adapted" logic adopted by nonprofit organizations in different stages of orchestration, constituting a useful reference for future nonprofit organizations orchestrating innovation networks.

Moreover, this study provides practical insights into the responses to global public health emergencies. Our systematic analysis of the global innovation network of the COVID-19 vaccine has shed light on how innovation networks can be efficiently led in the context of a global pandemic, as the COVAX program provides useful lessons on responding to a borderless health crisis. Due to the transnational nature of the COVID-19 outbreak, global collaboration became particularly critical, especially in achieving widespread vaccination, as only a global immunization barrier could achieve the effective containment of virus mutation and transmission, underscoring the centrality of the COVAX Program in the global health security landscape. However, COVAX is built on intricate geopolitics and lacks transparency in collaboration and decision-making, so equity issues have not been widely addressed in its implementation [11]. Examples include the reluctance of private companies to share knowledge about vaccines, over-reliance on voluntary donations, or weak manufacturing capacity in low- and middle-income countries [38]. Nevertheless, COVAX has managed to bring together the joint efforts of States, international organizations and civil society. A wide range of network resources are accessed and utilized by constructing and orchestrating global innovation networks. This study, however, is based on the theory of network orchestration and distills a theoretical model of COVAX orchestrating a global innovation network, which could be useful for similar global health crises that may arise in the future. By reviewing the series of mechanisms of action undertaken by COVAX to promote equity, we believe that multiple efforts are still needed in practice, including: ensuring the ideological unity of the participants and the establishment of a reliable structure of political participation, increasing the activeness of the participants on all sides through social media or other avenues, and leveraging the support of the participants of the private organizations in order to gain access to the supply of the vaccine and its effective distribution, among other things. Furthermore, we have deeply explored how to achieve value cocreation among multi-stakeholders in a network and, in particular, how nonprofit leadership teams can strategically orchestrate networks when facing key challenges in various stages. Taken together, the findings of this study can therefore provide strategic guidance in future global public health crises, enhancing the global community's ability to respond to them.

### 6.2 Limitations and prospects

Despite the research strengths of using longitudinal single-case studies in exploring the process of innovation network formation in nonprofit organizations, there are some shortcomings. First, the case in this paper was born during the COVID-19 pandemic, when network actions were relatively urgent and targeted, therefore, the selection of the COVID-19 vaccine global innovation network orchestration requires caution regarding the generalizability of its findings. Second, the individual cases themselves have inherent limitations, and this paper only analyses the case of the nonprofit organization COVAX, making it difficult to extrapolate the full landscape of innovation networks formed by nonprofit organizations. Therefore, future studies can select nonprofits in other contexts for comparison to increase the comprehensiveness of such studies. Finally, due to the specificity of the case, certain interview information was not available for this paper, and the analysis could only be conducted by analyzing public interview and the official websites of some of the leaders, which may have missed heterogeneous information. Therefore, subsequent studies can add interview information to explore the issue in depth.

## Data availability statement

Data will be made available on request.

## CRediT authorship contribution statement

**Hongming Xie:** Methodology, Investigation, Funding acquisition, Formal analysis, Data curation, Conceptualization. **Manman Guo:** Writing – review & editing, Writing – original draft, Visualization, Validation, Supervision, Software, Resources, Project administration. **Yingnan Yang:** Supervision, Writing – review & editing.

## Declaration of competing interest

The authors declare that they have no known competing financial interests or personal relationships that could have appeared to influence the work reported in this paper.

## Figures and Tables

**Fig. A1:**
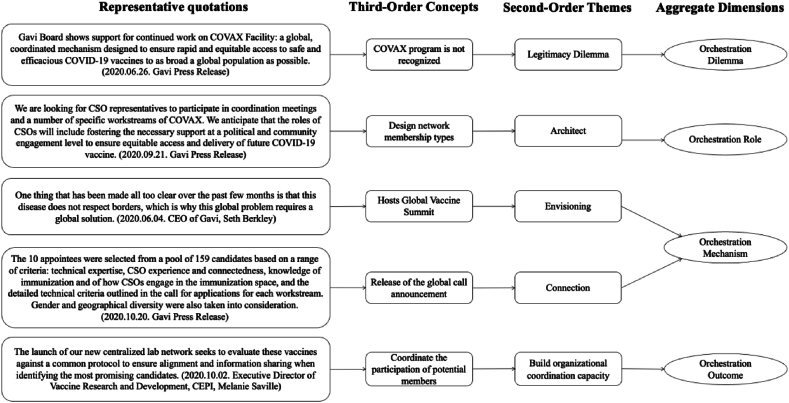
Coding of the COVID-19 Vaccine Global Innovation Network Construction Phase.

**Fig. A2:**
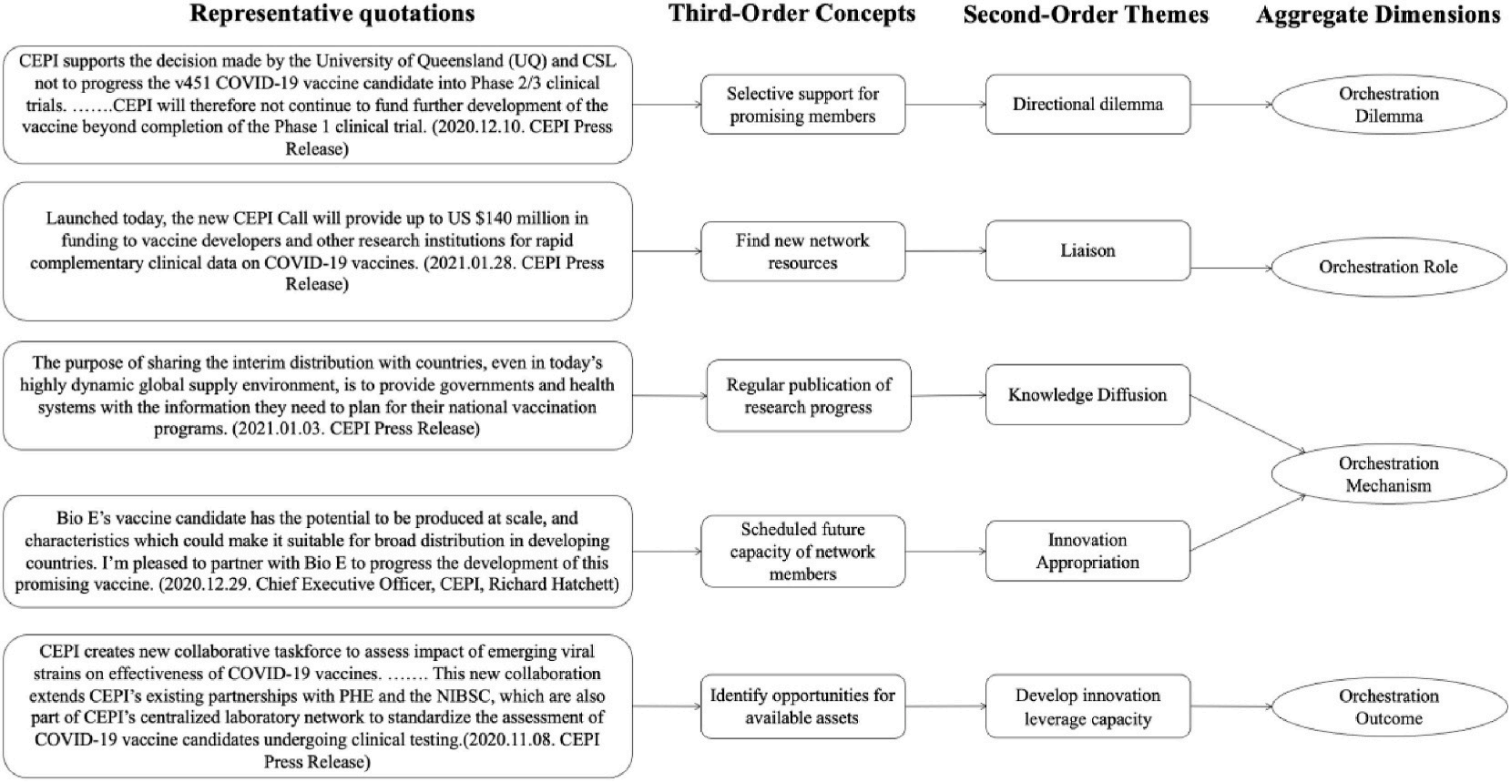
Coding of the COVID-19 Vaccine Global Innovation Network Operation Phase.

**Fig. A3:**
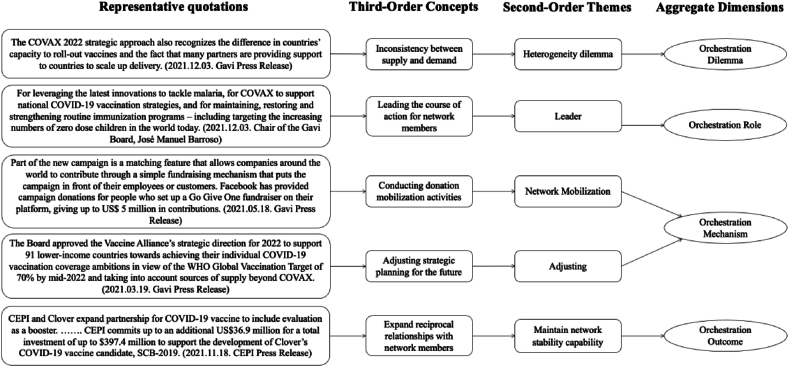
Coding of the COVID-19 Vaccine Global Innovation Network Maturity Phase.
